# Interventricular septal fat-containing mass most consistent with cardiac lipoma in an adult with tuberous sclerosis complex

**DOI:** 10.1016/j.radcr.2026.09.027

**Published:** 2026-10-13

**Authors:** Saubhagya Dhakal, Paulo Savoia, Nikahat Yasmine, Manisha Sharma

**Affiliations:** a Department of Radiology, University of Iowa Hospitals & Clinics, Iowa City, IA, USA; b Department of Pediatrics, University of Connecticut, Hartford, CT, USA

**Keywords:** Tuberous sclerosis complex, Cardiac lipoma, Interventricular septum, Myocardial fatty foci, Lymphangioleiomyomatosis, Angiomyolipoma

## Abstract

Tuberous sclerosis complex (TSC) is a multisystem genetic disorder in which cardiac rhabdomyomas and myocardial fatty foci are recognized cardiac manifestations, whereas discrete cardiac lipoma-like masses are uncommon. We report a 42-year-old woman with genetically and clinically confirmed TSC who underwent contrast-enhanced chest CT for surveillance of pulmonary lymphangioleiomyomatosis. CT demonstrated a well-circumscribed homogeneous macroscopic-fat attenuation mass centered in the mid-to-apical interventricular septum, measuring 2.8 × 2.2 × 2.0 cm with mean attenuation of approximately −92 HU and mild contour bulging toward the right ventricular cavity. The discrete mass-like morphology and homogeneous macroscopic fat favored cardiac lipoma over a typical TSC-associated myocardial fatty focus, although definitive distinction remains limited in the absence of dedicated cardiac MRI or histopathologic confirmation. This case emphasizes the imaging differential of fat-containing myocardial lesions in adults with TSC and the importance of integrating lesion morphology, size, tissue composition, and mass effect rather than relying on septal location alone.

## Introduction

Tuberous sclerosis complex (TSC) is a genetic multisystem disorder caused by pathogenic variants in the TSC1 or TSC2 genes, with variable involvement of the brain, kidneys, lungs, heart, skin, and other organs [1]. Major manifestations include cortical tubers and radial migration lines, renal angiomyolipomas, pulmonary lymphangioleiomyomatosis (LAM), and cardiac rhabdomyomas [1].

Cardiac rhabdomyoma is the characteristic cardiac tumor associated with TSC. It is most frequently identified during fetal life or infancy, is often multiple, and commonly involves the ventricular myocardium or interventricular septum [2,3]. Many rhabdomyomas undergo spontaneous regression during childhood [2,3].

In adults with TSC, myocardial fatty foci are increasingly recognized on CT and typically appear as small oval or linear intramyocardial areas of macroscopic fat without substantial mass effect [4]. Discrete cardiac lipomas, in contrast, are considerably less commonly reported in association with TSC [[5], [6], [7]].

We present an adult woman with extensive multisystem manifestations of TSC and a well-circumscribed macroscopic-fat attenuation mass centered in the interventricular septum. The principal educational value of this case is the imaging distinction between a discrete cardiac lipoma-like mass, TSC-associated myocardial fatty foci, and the more familiar cardiac rhabdomyoma.

### Case presentation

A 42-year-old woman with genetically and clinically confirmed tuberous sclerosis complex underwent contrast-enhanced chest CT for surveillance of known pulmonary lymphangioleiomyomatosis. Her medical history was notable for TSC, pulmonary LAM, seizures, and hematuria in the setting of extensive bilateral renal angiomyolipomas. She also had a family history of TSC. She denied chest pain, dyspnea, palpitations, syncope, or other cardiovascular symptoms. There was no history of cardiac surgery or invasive cardiac intervention.

Contrast-enhanced chest CT demonstrated innumerable bilateral diffusely distributed thin-walled pulmonary cysts with preserved intervening lung parenchyma, consistent with pulmonary LAM (Fig. 1). No pneumothorax, pleural effusion, or chylothorax was identified.

**Fig. 1:**
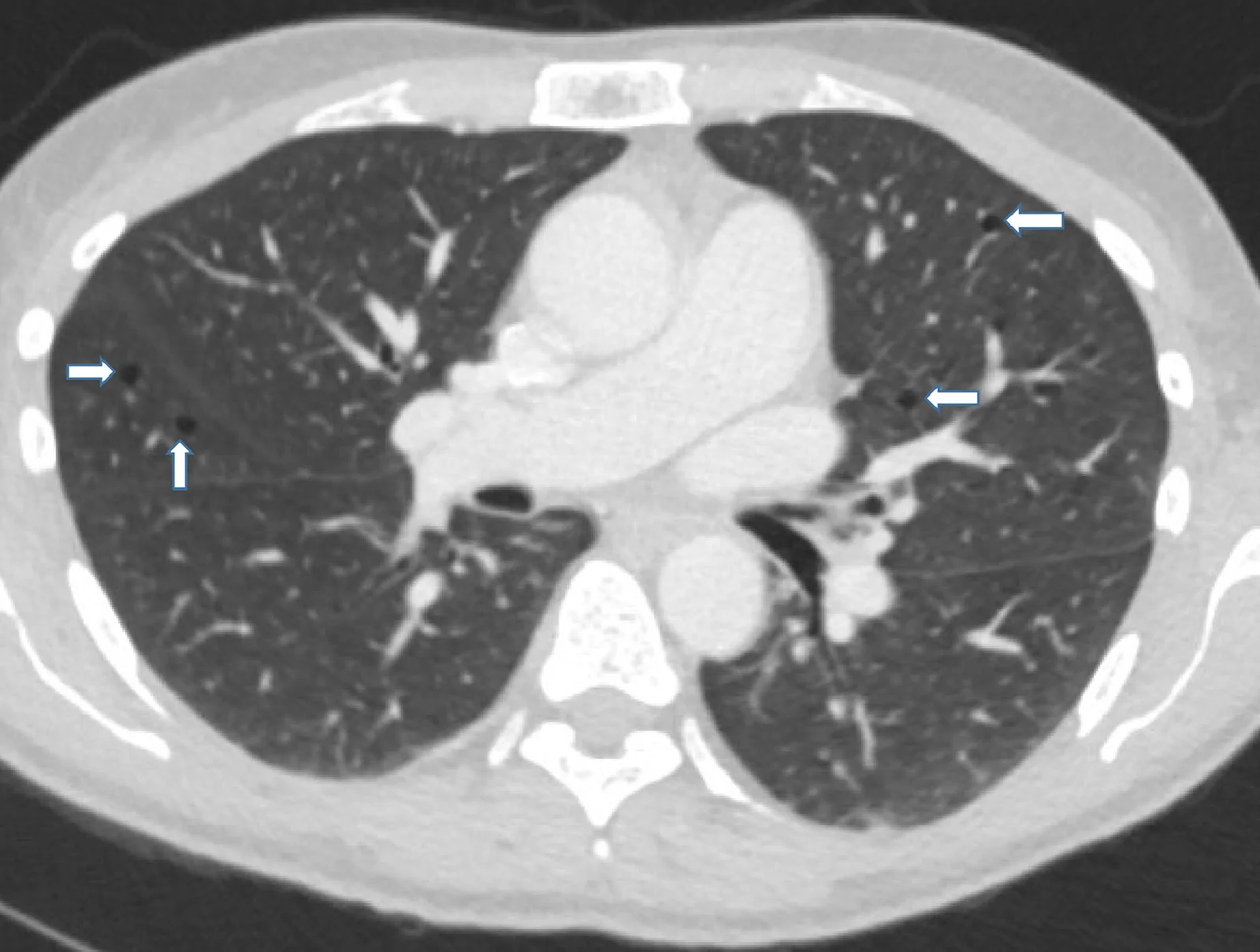
Pulmonary lymphangioleiomyomatosis in tuberous sclerosis complex. Axial lung-window chest CT image demonstrates innumerable bilateral diffusely distributed thin-walled pulmonary cysts with preserved intervening lung parenchyma, consistent with lymphangioleiomyomatosis.

An incidental well-circumscribed homogeneous fat-attenuation mass was identified within the mid-to-apical interventricular septum on axial contrast-enhanced chest CT (Fig. 2). The lesion measured 2.8 × 2.2 × 2.0 cm. A circular region of interest was placed within the homogeneous fatty portion of the lesion while avoiding its margins and adjacent myocardium as much as technically feasible, yielding a mean attenuation of approximately −92 HU and confirming macroscopic fat. The lesion produced mild contour bulging toward the right ventricular cavity without significant intracavitary extension. No internal enhancing soft-tissue component, calcification, or invasive feature was identified.

**Fig. 2:**
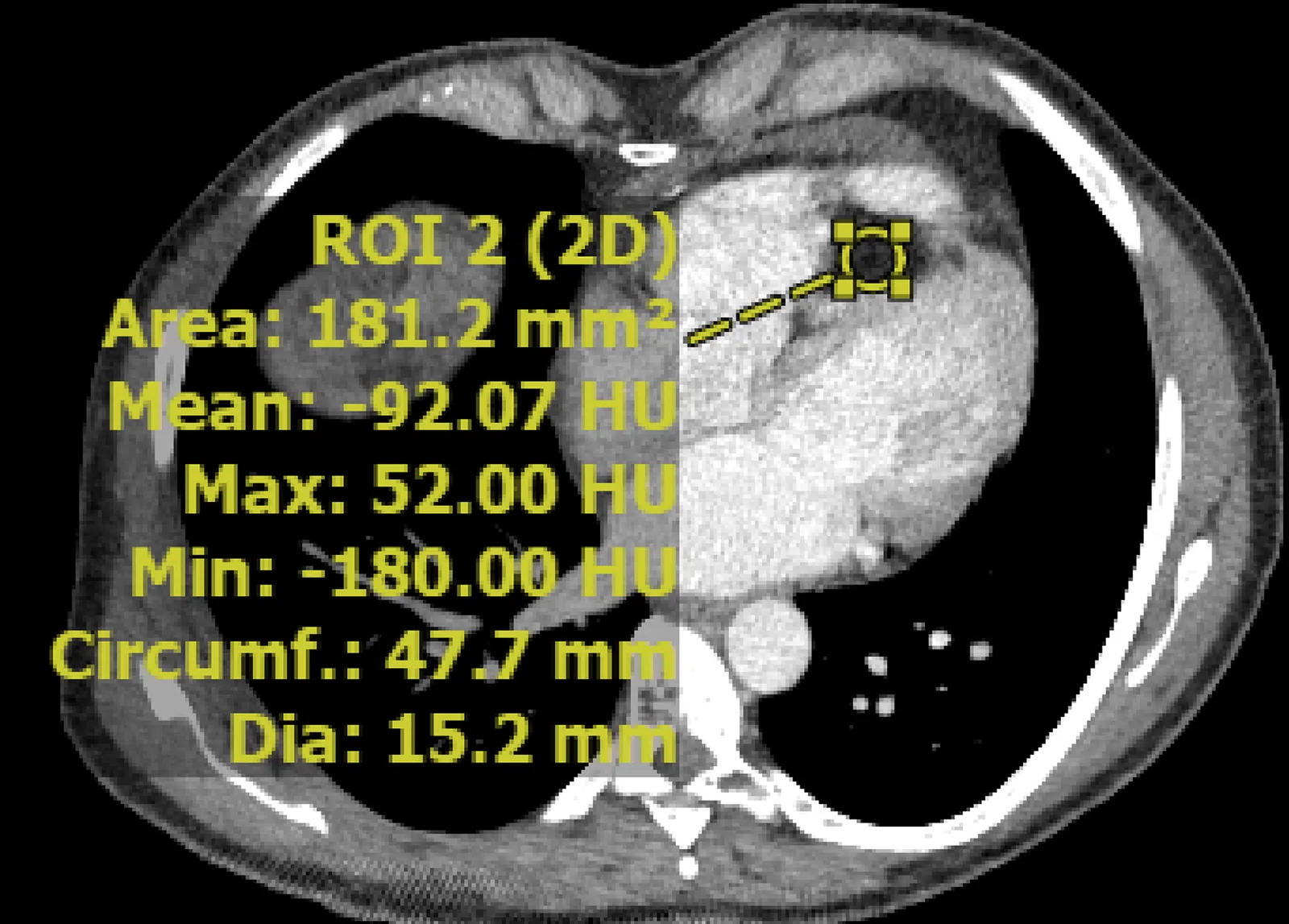
Interventricular septal fat-containing mass most consistent with cardiac lipoma. Axial contrast-enhanced chest CT image demonstrates a well-circumscribed homogeneous fat-attenuation mass centered within the mid-to-apical interventricular septum. A region of interest placed within the lesion demonstrates mean attenuation of approximately −92 HU, confirming macroscopic fat. The lesion measures 2.8 × 2.2 × 2.0 cm and produces mild contour bulging toward the right ventricular cavity without significant intracavitary extension.

The discrete mass-like morphology, well-defined margins, homogeneous macroscopic-fat attenuation, and mild contour effect favored cardiac lipoma rather than the typically smaller and non-mass-like myocardial fatty foci described in adults with TSC. Nevertheless, in the absence of dedicated cardiac MRI or histopathologic confirmation, the lesion was considered most consistent with cardiac lipoma on imaging rather than definitively proven.

Electrocardiography was normal, and no arrhythmia or clinical evidence of cardiac dysfunction was reported. Dedicated transthoracic echocardiographic findings were not available for evaluation of the incidentally detected lesion.

No prior chest CT adequately demonstrating the lesion and no dedicated cardiac MRI were available for comparison; therefore, long-term stability of the interventricular septal lesion could not be established. Histopathologic confirmation was not available. No lesion-directed management or intervention was documented in the available clinical record, and subsequent clinical or imaging follow-up specifically addressing the cardiac lesion was also not available. Laboratory results specifically relevant to characterization of the cardiac lesion were not available in the source record used for manuscript preparation.

Contrast-enhanced abdominal CT demonstrated extensive bilateral fat-containing renal angiomyolipomas involving much of the renal parenchyma (Figs. 3A and B). There was no active renal hemorrhage at the time of imaging.

**Fig. 3:**
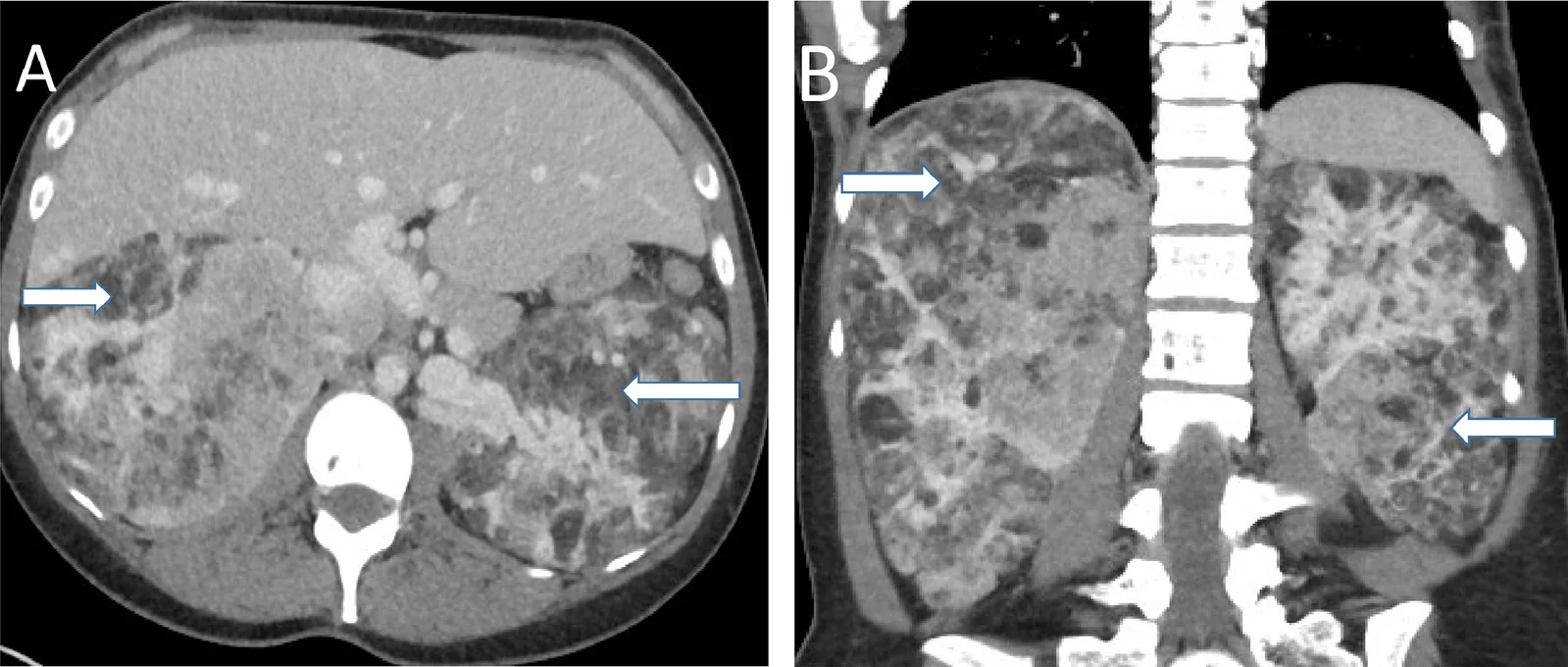
Extensive bilateral renal angiomyolipomas. (A) Axial contrast-enhanced abdominal CT image demonstrates extensive bilateral fat-containing renal angiomyolipomas involving much of the renal parenchyma. (B) Coronal contrast-enhanced abdominal CT image demonstrates the bilateral extent and multifocal distribution of the renal angiomyolipomas.

Brain MRI demonstrated cortical and subcortical T2/FLAIR hyperintense abnormalities associated with TSC, including lesions compatible with cortical tubers and/or radial migration lines (Fig. 4).

**Fig. 4:**
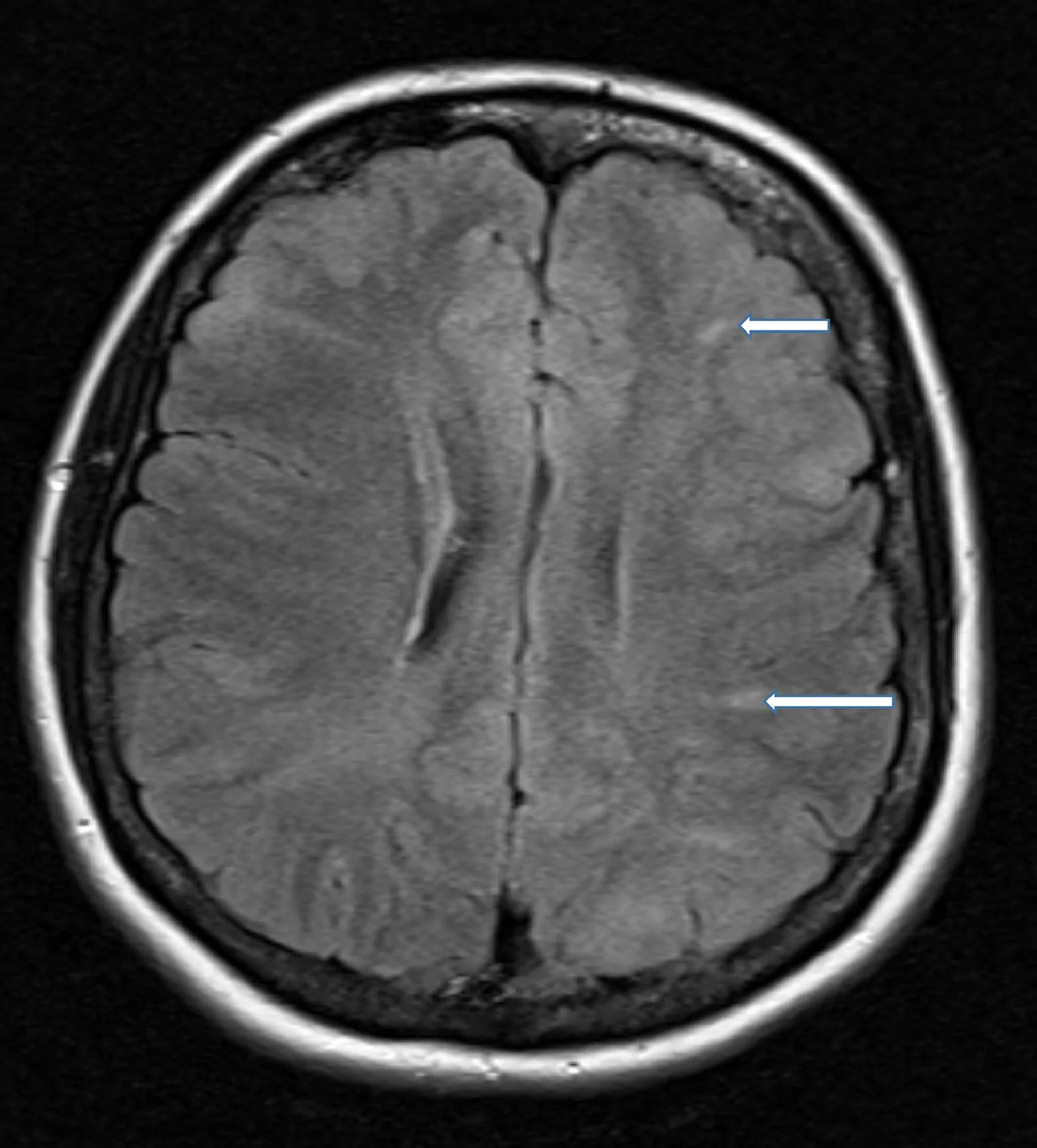
Central nervous system manifestations of tuberous sclerosis complex. Axial fluid-attenuated inversion recovery brain MRI demonstrates cortical and subcortical hyperintense abnormalities associated with tuberous sclerosis complex, including lesions compatible with cortical tubers and/or radial migration lines.

Collectively, the pulmonary LAM, bilateral renal angiomyolipomas, and characteristic central nervous system abnormalities established extensive multisystem involvement by TSC. The principal imaging finding relevant to this report, however, was the discrete interventricular septal macroscopic-fat mass.

## Discussion

Tuberous sclerosis complex is a multisystem genetic disorder characterized by dysregulation of mammalian target of rapamycin signaling and the development of hamartomatous lesions affecting multiple organs [1,7,8]. Cardiac rhabdomyomas are the characteristic cardiac tumors associated with TSC and are typically recognized prenatally or during infancy [[1], [2], [3]].

Although the interventricular septum is a common location for rhabdomyoma, the lesion in the present patient differed substantially from a typical rhabdomyoma in both patient age and tissue composition. The lesion was solitary, sharply circumscribed, homogeneous, and demonstrated unequivocal macroscopic-fat attenuation of approximately −92 HU without an enhancing soft-tissue component. These imaging characteristics are not typical of cardiac rhabdomyoma.

A more challenging distinction is between cardiac lipoma and the myocardial fatty foci increasingly recognized in adults with TSC. In a prior CT series, myocardial fatty foci were reported in up to approximately 50% of adults with TSC [4]. These foci are typically small, oval or linear intramyocardial areas of fat attenuation and generally lack appreciable mass effect [4]. Septal involvement may occur, and therefore location alone does not reliably distinguish a myocardial fatty focus from a cardiac lipoma.

Importantly, universally accepted imaging size thresholds or morphologic criteria that definitively separate myocardial fatty foci from cardiac lipomas in patients with TSC have not been established. Consequently, the distinction may remain partly subjective, particularly in the absence of dedicated cardiac MRI or histopathologic confirmation.

In the present case, the lesion measured 2.8 cm in maximum dimension and was solitary, sharply circumscribed, homogeneous in fat attenuation, and distinctly mass-like, with mild contour bulging toward the right ventricular cavity. These features favor cardiac lipoma over a typical TSC-associated myocardial fatty focus. Nevertheless, because dedicated cardiac MRI and histopathologic confirmation were not available, we consider the diagnosis imaging-based and appropriately describe the lesion as most consistent with cardiac lipoma.

Burt et al. described multiple myocardial fatty foci together with a larger subendocardial lipoma involving the interventricular septum in a patient with TSC, illustrating that these two fat-containing cardiac manifestations may coexist and may represent morphologically distinct findings [7]. Cardiac lipoma in adults with TSC has also been described with histopathologic and cardiac MRI characterization [5,6].

Accordingly, the educational contribution of the present case is not the assertion that cardiac lipoma has never previously been reported in TSC. Rather, this case illustrates a practical imaging challenge encountered in adults with TSC: distinguishing a discrete macroscopic-fat cardiac mass from the substantially more commonly recognized myocardial fatty foci. Recognition of comparatively large size, sharply defined margins, homogeneous macroscopic-fat composition, and a true mass-like configuration may favor lipoma, while the absence of histopathology or dedicated cardiac MRI requires appropriate diagnostic caution.

Cardiac lipomas are benign tumors composed predominantly of mature adipose tissue. On CT, they characteristically appear as well-defined homogeneous lesions with attenuation similar to subcutaneous fat [9]. Cardiac MRI can provide further tissue characterization by demonstrating high T1 signal with suppression on fat-saturated sequences. In the current case, the homogeneous −92 HU attenuation, absence of enhancing soft tissue, lack of invasive characteristics, and discrete mass-like morphology strongly supported a lipomatous lesion.

Clinical manifestations of cardiac lipoma depend on lesion size and location and may include arrhythmia, conduction disturbance, ventricular inflow or outflow obstruction, valvular dysfunction, coronary compression, or pericardial effusion [9]. In the present patient, the lesion caused only mild contour bulging toward the right ventricular cavity without significant intracavitary extension. The patient had no cardiovascular symptoms, and electrocardiography was normal.

Pulmonary LAM, extensive bilateral renal angiomyolipomas, and characteristic central nervous system abnormalities established the patient's extensive multisystem TSC phenotype. These associated findings provide important disease context but remain secondary to the principal educational feature of this case: the discrete interventricular septal macroscopic-fat mass and its distinction from other cardiac manifestations of TSC.

The principal limitations of this report are the absence of histopathologic confirmation, dedicated cardiac MRI, echocardiographic characterization, and prior or subsequent imaging establishing stability. Therefore, although the CT morphology strongly favors cardiac lipoma, definitive separation from an unusually large or mass-like myocardial fatty focus cannot be established with absolute certainty.

## Conclusion

In adults with tuberous sclerosis complex, macroscopic myocardial fat may represent the relatively common myocardial fatty foci of TSC or, less commonly, a discrete cardiac lipoma. A comparatively large, sharply circumscribed, homogeneous macroscopic-fat attenuation lesion with a true mass-like configuration favors lipoma over a typical myocardial fatty focus. However, definitive distinction may remain limited in the absence of dedicated cardiac MRI or histopathologic confirmation. Recognition of these overlapping entities is important for appropriate characterization of incidental cardiac findings in patients with TSC and for avoiding diagnostic overstatement.

## Ethics statement

This single-patient, de-identified case report did not require institutional review board approval according to institutional policy.

## Patient consent

Written informed consent was obtained from the patient for publication of this case report and all accompanying images. All clinical and imaging information has been de-identified, and no personally identifiable information is included in the manuscript or figures.

## Appendix Supplementary materials

Image, application 1
