# Supplementary material for: Interventricular septal fat-containing mass most consistent with cardiac lipoma in an adult with tuberous sclerosis complex
Source: Radiol Case Rep. 2026 Oct 13;21(12):6519–23. doi: 10.1016/j.radcr.2026.09.027 (PMC13684998; doi:10.1016/j.radcr.2026.09.027)
Supplement: Supplementary file 1 [file mmc1.docx]

**CARE Checklist for Case Reports**

**Manuscript title:** Interventricular Septal Cardiac Lipoma in an Adult With Tuberous Sclerosis Complex

| **CARE checklist item** | **Information included in manuscript** | **Manuscript location** |
| --- | --- | --- |
| **1. Title** | The title identifies the key diagnosis/clinical finding and indicates that the report concerns an adult patient with tuberous sclerosis complex. | Title page |
| **2. Keywords** | Tuberous sclerosis complex; cardiac lipoma; interventricular septum; lymphangioleiomyomatosis; angiomyolipoma. | Title page |
| **3a. Abstract – Introduction** | Brief background on TSC, the usual association with cardiac rhabdomyoma, and the unusual nature of cardiac lipoma. | Abstract |
| **3b. Abstract – Case presentation** | A 42-year-old woman with genetically and clinically confirmed TSC underwent chest CT surveillance for LAM. CT demonstrated a 2.8 × 2.2 × 2.0 cm homogeneous fat-attenuation interventricular septal lesion with mean attenuation of −92 HU, most consistent with cardiac lipoma. Coexisting LAM, bilateral renal AMLs, and cortical tubers were present. | Abstract |
| **3c. Abstract – Conclusion** | Recognition of a discrete fat-containing cardiac mass in TSC is important to distinguish cardiac lipoma from rhabdomyoma and myocardial fatty foci. | Abstract |
| **4. Introduction** | Provides background on TSC, cardiac rhabdomyoma, myocardial fatty foci, and the rarity of discrete cardiac lipoma in TSC. | Introduction |
| **5. Patient information** | A 42-year-old woman with genetically and clinically confirmed TSC, family history of TSC, seizures, and hematuria. | Case presentation |
| **6. Clinical findings** | The patient had no chest pain, dyspnea, palpitations, syncope, or other cardiovascular symptoms. Electrocardiography was normal. | Case presentation |
| **7. Timeline** | The patient had established TSC and underwent chest CT for surveillance of known pulmonary LAM. Imaging also demonstrated cardiac, renal, and cerebral manifestations of TSC. | Case presentation; Table 1 |
| **8a. Diagnostic assessment – Methods** | Contrast-enhanced chest CT, contrast-enhanced abdominal CT, and brain MRI with axial FLAIR imaging were reviewed. | Case presentation |
| **8b. Diagnostic assessment – Challenges** | No cardiac MRI or histopathologic confirmation was available. Therefore, the cardiac lesion was diagnosed on imaging as most consistent with cardiac lipoma. | Discussion |
| **8c. Diagnostic assessment – Diagnosis** | Well-circumscribed homogeneous macroscopic fat-containing lesion in the mid-to-apical interventricular septum, measuring 2.8 × 2.2 × 2.0 cm with mean attenuation of −92 HU, most consistent with cardiac lipoma. | Case presentation; Fig. 2 |
| **8d. Diagnostic assessment – Prognosis** | No cardiac symptoms, arrhythmia, ECG abnormality, right ventricular outflow tract obstruction, pericardial effusion, or invasive imaging features were identified. | Case presentation; Discussion |
| **9. Therapeutic intervention** | No surgical or invasive cardiac intervention had been performed. | Case presentation |
| **10a. Follow-up and outcomes** | The patient remained clinically without reported cardiac symptoms at the time of case assessment. | Case presentation |
| **10b. Follow-up adherence and tolerability** | Not applicable because no specific cardiac intervention was performed. | Not applicable |
| **10c. Adverse and unanticipated events** | No adverse cardiac events, arrhythmia, obstruction, or pericardial effusion were reported. | Case presentation |
| **11a. Discussion – Strengths and limitations** | The report highlights a rare imaging-diagnosed interventricular septal cardiac lipoma-like lesion in an adult with multisystem TSC. Limitations include lack of cardiac MRI and histopathologic confirmation. | Discussion |
| **11b. Discussion – Relevant literature** | The findings are discussed in relation to TSC-associated rhabdomyomas, myocardial fatty foci, and previously reported cardiac lipomas. | Discussion; References |
| **11c. Discussion – Rationale for conclusions** | The diagnosis was supported by discrete mass-like morphology, homogeneous macroscopic fat attenuation, absence of enhancing soft tissue, and lack of invasive features. | Discussion |
| **12. Patient perspective** | Not available. | Not applicable |
| **13. Informed consent** | Written informed consent for publication of the case report and accompanying images was obtained from the patient. | Patient Consent Statement |

**Table 1. Clinical and Imaging Timeline**

| Time point | Clinical and imaging findings |
| --- | --- |
| Prior history | Genetically and clinically confirmed tuberous sclerosis complex; family history of TSC; history of seizures and hematuria. |
| Chest CT surveillance | Contrast-enhanced chest CT performed for known pulmonary lymphangioleiomyomatosis surveillance. |
| Chest CT findings | Innumerable bilateral diffuse thin-walled pulmonary cysts consistent with LAM; discrete 2.8 × 2.2 × 2.0 cm mid-to-apical interventricular septal fat-containing mass with mean attenuation of −92 HU. |
| Cardiac assessment | No chest pain, dyspnea, palpitations, syncope, arrhythmia, or ECG abnormality. No right ventricular outflow tract obstruction or pericardial effusion. |
| Abdominal CT findings | Extensive bilateral renal angiomyolipomas involving much of the renal parenchyma; no active renal hemorrhage. |
| Brain MRI findings | Axial FLAIR MRI demonstrated elongated cortical/subcortical hyperintense lesions consistent with cortical tubers. |
| Management at case assessment |  |
